# Levels and factors of knowledge about the related health risks of exposure to secondhand smoke among medical students: A cross-sectional study in Jeddah, Saudi Arabia

**DOI:** 10.18332/tid/128317

**Published:** 2020-10-30

**Authors:** Sami H. Alzahrani

**Affiliations:** 1 Department of Family Medicine, Faculty of Medicine, King Abdulaziz University, Jeddah, Saudi Arabia

**Keywords:** secondhand smoke, passive smoking, exposure, knowledge, awareness

## Abstract

**INTRODUCTION:**

Secondhand smoke (SHS) appears to be an insidious public health issue in Saudi Arabia, with a high percentage of people being reportedly exposed. In an attempt to raise awareness about this issue, we explored medical students’ level of knowledge about SHS risks, as well as their levels of exposure to SHS and the correlation between knowledge and exposure.

**METHODS:**

A cross-sectional study was conducted in February 2020 at the Faculty of Medicine, King Abdulaziz University, Jeddah, Saudi Arabia. An online, modified version of a validated questionnaire was distributed among 2nd to 6th year medical students. The questionnaire mainly explored the following items: 1) exposure level to SHS; 2) impact of SHS exposure; and 3) knowledge about SHS related health risks, with calculation of a knowledge score (range: 0–8). Adequate knowledge was defined as a score ≥5 (median = 5), and associations with sociodemographic and lifestyle factors and exposure to SHS were analyzed using chi-squared and independent t-test, as appropriate.

**RESULTS:**

Of a total 416 participants, 65.0% declared having one or more smoking persons among acquaintances, 26.4% being exposed to SHS at home, and 40.1% exposed to parental smoking during childhood. Further, 79.8% reported being regularly exposed to SHS in public places. Majority of participants reported significant discomfort (53.2%) and physical symptoms (98.3%) subsequent to SHS exposure. Among the list of 8 health risks, ear infection in children (28.1%), heart attack in children (37.5%), and cognitive deficit (47.8%) were the least frequently identified. Adequate knowledge was found among 57.5% of the participants, and was higher among females and lower among participants living with their friends, compared to their counterparts (p<0.05). No association of knowledge level was observed with the parameters of exposure to SHS and poor discomfort feeling.

**CONCLUSIONS:**

The level of knowledge of medical students about health risks related to passive smoking is inadequate, while their exposure to SHS in public places is still substantial. This urges for the reinforcement of tobacco control strategies and highlights the great interest for medical colleges to implement effective educational interventions by improving their curricula regarding the risks of SHS and the benefits of smoking cessation.

## INTRODUCTION

For a long time, smoking has been identified as a major risk factor of multiple illnesses and mortality worldwide^1^, representing one of the greatest health epidemics in the twentieth century. Smokers are prone to a diverse array of negative consequences as indicated by the susceptibility to acute and longterm deleterious illnesses such as respiratory diseases, cancer, coronary heart disease, and deterioration in self-reported health, with subsequent school/work absenteeism and mortality^2,3^. Such consequences are complicated by the substantial prevalence of smoking. For example, in Saudi Arabia, the prevalence of smoking ranges between 13% and 38% among males, and this is supported by the significant rise of tobacco sales relative to the population size^4,5^. The pooled prevalence among college students has been estimated at 17%, and it is higher in males than in females (26% versus 5%, respectively)^6^. Smoking has been a significant risk factor for cardiovascular diseases in the kingdom, and it is a forthcoming alarming threat for other tobacco-related illnesses, such as chronic obstructive pulmonary disease^7^.

However, the impact of the toxic effects of tobacco smoking is not only limited to active smokers, but also includes individuals exposed to secondhand smoke (SHS) or passive smokers. For instance, children of smoking parents are susceptible to short-term health risks, such as ear and respiratory diseases, as well as chronic diseases including inflammatory bowel disease and endothelial cell dysfunction^8,9^. Besides, the exposure to SHS has been associated with metabolic deficits, such as DNA and lipid oxidative damage. Additionally, individuals exposed to SHS have similar patterns of reduced antioxidant mechanisms as those observed in active smokers, such as reduced concentrations of vitamin C and vitamin A^10^. Consequently, SHS exposure is causally associated with lessened immune status, reduced lung functions, stroke, coronary heart disease, and other chronic diseases as in the case of active smoking^11^, and SHS-exposed children are more likely to experience leukemia, brain tumors, and lymphomas than nonexposed children^1,12^. Although the health risks of SHS are less frequently encountered than those in active smokers, the fact that the harmful effects are incurred even at very low doses indicates the lack of a specific safe threshold of exposure^13^. This emphasizes the need to address such a public health challenge.

The implications of these risks are significant, particularly in areas with a high prevalence of SHS exposure. In Saudi Arabia, despite a reinforced antismoking law banning smoking in public areas and explicitly stating the priority to protect non-smokers from exposure to tobacco smoke and emissions, particularly persons under 18 years of age^14^, the issue of SHS is still a national concern. A nationwide study contributing to the Global Youth Tobacco Survey (GYTS) revealed that three in ten adolescents are exposed to SHS at home, and about 37.5% of students aged 13–15 years are exposed to smoke outdoors^15^.

Therefore, the role of physicians in treating tobacco dependence and raising public awareness about the health risks associated with smoking and SHS is more than ever critical. It has been shown that brief advice can markedly increase cessation rates^16,17^. However, there are multiple challenges associated with appropriate counselling. These include lack of self-efficacy and self-confidence in counselling skills, lack of proficiency with the methods of cessation and the risks of SHS exposure, lack of allocated time, and the lack of adequate cessation services integrated in healthcare systems^18^.

These challenges are compounded by the attitudes and levels of knowledge regarding smoking and SHS exposure among healthcare workers, and several local studies indicate a deficit in medical education curricula in tobacco dependence treatment and tobacco related topics, notably in undergraduate years^18-20^. On the other hand, the prevalence of smoking among medical students locally ranged from 13% to 28.9%^21-23^, and there is evidence of the role of peers in Saudi colleges as predictors of future active smoking^5,24^.

Indeed, these figures are alarming since healthcare providers, including medical students, should have adequate knowledge of SHS and be highly aware of its importance to educate patients, smokers and the public. Thus, we aimed at providing insight into the gaps in knowledge about SHS among medical students, and profiling the healthiness of their lifestyle and academic environment.

The present study assessed the level of knowledge of medical students regarding SHS-related health risks, and secondarily explored the associated factors with knowledge, as well as sources of information. Furthermore, it investigated the levels of exposure to SHS in the students’ living and study environments and the impact of such exposure on somatic health and behavior.

## METHODS

### Population and setting

A cross-sectional study was conducted in February 2020 among medical students at the Faculty of Medicine, King Abdulaziz University, Jeddah, Saudi Arabia. All undergraduate medical students from second to sixth year were considered eligible to participate. Students aged <18 years or >30 years were not included. The study was approved by the Institutional Review Board of King Abdulaziz University (Institutional Review Board 22-1-19). After obtaining ethical approval for the research from KAU, the email addresses of the selected students were obtained from Academic Affairs. The questionnaire was administered online via the SurveyMonkey platform (SurveyMonkey Europe UC), and the web link was sent via email to all selected participants, including a statement for explicit and written consent to participate. In addition, a notification was attached about the study objectives and response confidentiality. Potential participants were advised that the study results would be involved in a statistical analysis and aimed at being published in a peer-reviewed journal. The datasets used and/or analyzed during the current study are available from the author on reasonable request and all original data are available from the Department of Family Medicine, KAU, Jeddah, Saudi Arabia.

### Sampling

Target sample size (N=332) was calculated to detect an unknown proportion (50%) of students with adequate knowledge about SHS risk factors out of 2410 eligible medical students, with 5% margin error and 95% confidence interval. This was calculated using Raosoft software (Raosoft, Seattle, WA, USA), available at: http://www.raosoft.com/samplesize.html. The target sample size was increased to N=480 to compensate for eventual non-response, assuming 70% response rate.

The target population was stratified by academic level (2nd, 3rd, …, year of studies) and a proportional allocation was used to randomly include participants based on the weighted percentage of students in each academic year. The sample sizes were: 25% (152/609) from 2nd year, 20% (98/491) from 3rd year, 21% (106/507) from 4th year, 17% (72/424) from 5th year, and 15% (57/379) from 6th year.

### Questionnaire structure

Permission was obtained from Lee et al.^25^ to use their questionnaire, as published in a study on SHS knowledge among hospital staff. An electronic, modified version of the questionnaire was adapted by not including the Fagerström test for nicotine dependence and some irrelevant (for Saudi Arabia) items such as alcohol consumption. The questionnaire covered five topics.

#### Parameters of exposure to SHS

These included: a number of smoking person categories among acquaintances including father, mother, brothers, sisters, friends, and other relatives, each accounting for one person-category; history of living with a smoking parent during childhood; places of SHS exposure, by answering the question ‘where are you usually exposed to SHS?’ with response options: at home – living room, at home – outside room, around the hospital, in the hospital, street corners, public places; and other exposure to SHS by any chance.

#### Impact and consequences of exposure to SHS

These included: perceived level of discomfort when exposed to SHS using a 5-point Likert-type discomfort scale (from ‘feels good’ to ‘painful’); any somatic symptoms (nose and eyes irritation, respiratory symptoms, chest discomfort, any other symptom); coping strategy (asked them to refrain from smoking, moved away from smoke, did nothing, or smoked together).

#### Awareness and knowledge about SHS health risks

This was ascertained by a question on SHS related health risks that included lung cancer, heart diseases, cognitive deficits, low birthweight, ear infections in children, heart attack to children, allergies in children, and asthma in children. Answers to the awareness and knowledge question were evaluated using a 5-point Likert-type scale of agreement (from ‘strongly agree’ to ‘strongly disagree’).

#### Sources of knowledge about SHS risks

This was ascertained by a question on the different sources of knowledge about SHS risks with options that included newspapers, TV programs, public service announcement, smoking cessation education, and acquaintances such as relatives and friends, with responses provided as ‘yes’ or ‘no’.

#### Opinions regarding smoking prohibition in public places

These included the need to make the prohibition compulsory, leaving it to the individual’s personal conscience, or no prohibition at all.

Additionally, sociodemographic characteristics such as age, gender, marital status, GPA etc., and clinical and lifestyle data such as physical activity, history of chronic diseases, and smoking status, were collected.

### Statistical analysis

After completion of the questionnaire, results were downloaded in a comma-separated values file format. The dataset was converted into an SPSS database (SPSS version 21.0 for Windows, SPSS Inc., Chicago, IL, USA). Descriptive statistics were used to summarize participants’ sociodemographic and lifestyle characteristics as well as the pattern of answering to the different parts of the questionnaire. Knowledge subscale (8 items) was analyzed for reliability by calculating Cronbach’s alpha. Knowledge score (range: 0–8) was calculated as the number of health risks that were correctly identified by the participant, where ‘strongly agree’ or ‘agree’ were considered as a correct identification. The distribution of the variable (knowledge score) was analyzed using Kolmogorov-Smirnov and Shapiro-Wilk tests. Given non-normal distribution of knowledge score (see results), knowledge level was dichotomized into adequate and inadequate knowledge, using a knowledge score ≥median to define adequate knowledge. Thus, the association of knowledge level (the dependent variable) with the independent variables including sociodemographic and lifestyle factors, parameters of exposure to SHS, and discomfort feeling regarding exposure and attitude towards prohibition of smoking in public places were analyzed using a chi-squared test for categorical variables, and independent t-test for continuous variables such as age. Multivariate binary regression was carried out using the ‘enter’ method to analyze the independent factors of adequate knowledge; results are presented as odds ratio (OR) with 95% confidence interval (95% CI). In such a regression model, the level of knowledge was the dependent variable and the significantly associated factors were entered as independent variables. Secondarily, we analyzed the association of perceived level of discomfort with parameters of exposure to SHS including number of smoking persons among acquaintances and number of exposure places; we analyzed both discomfort score (range: 0–4) and percentage of participants with high level of discomfort (≥3), using Kruskal-Wallis (nonparametric) test and chi-squared test, respectively. A p value <0.05 was considered to reject the null hypothesis.

## RESULTS

### Participants’ characteristics

A total of 416 medical students replied to the questionnaire (response rate: 86.7%); mean (SD) age 21.75 (1.60) years, 56.0% females, and 97.8% single. Distribution by academic level ranged from 10.3% for 3rd year to 26.9% for 6th year, and the majority had high (38.9%) or average (56.0%) grade point average (GPA). Clinical and lifestyle characteristics showed that 23.1% were overweight and 13.9% obese, 50.7% with low level of physical activity, and 12.3% having a chronic disease. Smoking status showed 13.9% active smokers and 4.6% quitters, with the majority of active smokers (63.8%) consuming >10 cigarettes daily (Table 1).

**Table 1:** Characteristics of medical students of the Faculty of Medicine, King Abdulaziz University (N=416)

*Characteristics*	*Category*	*n*	*%*
**Sociodemographic**			
**Age** (years), mean ± SD			21.75 ± 1.60
**Gender**	Male	183	44.0
	Female	233	56.0
**Marital status**	Single	407	97.8
	Married	9	2.2
**Nationality**	Saudi	398	95.7
	Non-Saudi	18	4.3
**Parents’ marital status**	Married	341	82.0
	Divorced	33	7.9
	Widow	42	10.1
**Rank in siblings**	Eldest	88	21.2
	Not the eldest	328	78.8
**Monthly family income** (SAR)	<3000	13	3.1
	3000–10000	77	18.5
	>10000	326	78.4
**Residence**	Jeddah	383	92.1
	Other	33	7.9
**Housing**	Rental	316	76.0
	Own house	83	20.0
	Students dormitory	17	4.1
**Living modality**	Alone	381	91.6
	With family	7	1.7
	With friends	28	6.7
**Academic year**	2nd	94	22.6
	3rd	43	10.3
	4th	94	22.6
	5th	73	17.5
	6th	112	26.9
**GPA**	≥4.5	162	38.9
	3.5–4.49	233	56.0
	2.5–3.49	21	5.0
**Lifestyle and clinical**			
**BMI** (kg/m^2^)	Underweight (<18.5)	49	11.8
	Normal (18.5–24.9)	213	51.2
	Overweight (25.0–29.9)	96	23.1
	Class I obesity (30.0–34.9)	38	9.1
	Class II obesity (35.0–39.9)	9	2.2
	Class III obesity (≥40.0)	11	2.6
**Physical activity** (times per week)	0	124	29.8
	1	87	20.9
	2–3	40	9.6
	>3	165	39.7
**Chronic illness**	No	365	87.7
	Yes	51	12.3
	Asthma	20	4.8
	Diabetes	11	2.6
	Chronic neurological disease	6	1.4
	Hypertension	3	0.7
	Not specified	13	3.1
**Smoking status**	Non-smoker	339	81.5
	Quitter (>6 months)	19	4.6
	Smoker	58	13.9
**Daily cigarettes** (among smokers)	0–3	13	3.1 (22.4)
	4–10	8	1.9 (13.8)
	11–20	18	4.3 (31.0)
	>20	7	1.7 (12.1)
	Not specified	12	2.9 (20.7)

SAR: 100 Saudi Riyal about 27 US$. GPA: grade point average. SD: standard deviation.

### Secondhand smoke exposure, consequences and coping strategies

In all, 75% of the participants declared having at least one smoking person among acquaintances, who was more frequently a relative (38.5%), a friend (34.6%), or the father (26.7%). Further, 40.1% reported exposed to parental smoking during childhood. Regarding exposure places, majority reported being exposed to SHS in public places (79.8%), while 26.4% reported being exposed at home, in room, and 24.0% outside room. On the other hand, 57.7% were regularly exposed to SHS in more than one place. Subsequent to exposure, 53.2% of the participants declared being very or extremely uncomfortable, 98.3% reported experiencing physical symptoms such as chest discomfort (27.9%), respiratory symptoms (27.6%) and nose and eye irritations (25.5%). The most frequently reported coping strategy regarding exposure to SHS was moving away from smoke (63.7%), having a passive attitude (21.4%), or asking smokers to refrain from smoking (8.2%) (Table 2).

**Table 2:** Assessment of secondhand smoke exposure consequences and coping strategies among medical students of the Faculty of Medicine, King Abdulaziz University (N=416)

*Type of exposure*	*Category*	*n*	*%*
**Smokers among social circle/ relatives^a^**	Nobody	104	25.0
	Yes (at least one person)	312	75.0
	Father	111	26.7
	Mother	24	5.8
	Brother	96	23.1
	Sister	24	5.8
	Relative	160	38.5
	Friend	144	34.6
**Number of persons smoking among acquaintances**	0	104	25.0
	1	165	39.7
	2	78	18.8
	3	46	11.1
	≥4	23	5.5
**Having a smoking parent during childhood**	No	249	59.9
	Yes	167	40.1
**Other exposure by chance/ place(s)^a^**	No	57	13.7
	Yes	359	86.3
	At home, in room	110	26.4
	At home, outside room	100	24.0
	Around the hospital	129	31.0
	In the hospital	23	5.5
	Public places	332	79.8
	Street corners	147	35.3
**Number of places with SHS exposure**	0–1	176	42.3
	2	118	28.4
	3	79	19.0
	≥4	43	10.3
**Feeling during exposure**	Good	14	3.4
	Not uncomfortable	67	16.1
	A bit uncomfortable	114	27.4
	Very uncomfortable	202	48.6
	Painful	19	4.6
**Symptoms arising from exposure^a^**	Nose and eyes irritation	106	25.5
	Respiratory symptoms	115	27.6
	Chest discomfort	116	27.9
	Child respiratory	15	3.6
	Any other symptom	186	44.7
**Coping strategy**	Asked them to refrain from smoking	34	8.2
	Moved away to avoid SHS	265	63.7
	Did nothing	89	21.4
	Smoked together	28	6.7

a More than one option possible.

### Knowledge about secondhand smoke risks and attitude to smoking prohibition in public places

Ear infection in children (28.1%), heart attack in children (37.5%), and cognitive deficit (47.8%) were the least frequently identified health risks of SHS among the list of eight; while lung cancer (84.9%), asthma in children (80.3%) and heart diseases (75.7%) were the most frequently identified (Table 3). Reliability testing of the knowledge subscale showed a Cronbach alpha of 0.763, indicating good internal consistency. Knowledge score showed a mean (SD) of 4.77 (2.01), median of 5, and range of 0–8; and normality testing showed KolmogorovSmirnov (statistic = 0.121, p<0.0001); Shapiro-Wilk (statistic = 0.956, p<0.0001) concluding to nonnormally distributed variable (results not presented). Consequently, 57.5% of the participants had adequate knowledge, which was defined as a knowledge score ≥5, the median being the cutoff. Acquaintances contributed to knowledge about SHS among 61.1% of the participants, while public service announcements and smoking cessation education contributed 44.0% and 38.9%, respectively. Majority of the participants (64.4%) thought that smoking should be strictly prohibited in public places while 9.4% advocated the right to smoke (Table 3).

**Table 3:** Knowledge about secondhand smoke risks and attitude regarding smoking prohibition in public places among medical students of the Faculty of Medicine, King Abdulaziz University (N=416)

*Parameter*	*Category*	*n*	*%*
**Awareness about SHS risks**	Yes	320	76.9
	No	96	23.1
		
**SHS-related health risks correctly identified**	Lung cancer	353	84.9
	Heart diseases	315	75.7
	Cognitive deficit	199	47.8
	Low birthweight	266	63.9
	Ear infection in children	117	28.1
	Heart attack in children	156	37.5
	Allergies in children	243	58.4
	Asthma in children	334	80.3
**Knowledge level**	Inadequate (score <5)	177	42.5
	Adequate (score ≥5)	239	57.5
**Knowledge source^a^**	Newspapers	37	8.9
	TV programs	157	37.7
	Public service announcement	183	44.0
	Smoking cessation education	162	38.9
	Acquaintances	254	61.1
**What do you think about prohibiting smoking in public places?**	Ignoring the right to smoke is unfair	39	9.4
	Better leave it to each individual’s personal conscience	109	26.2
	It should be regulated more strictly	268	64.4

a More than one option possible.

### Factors associated with knowledge about secondhand smoke risks and attitude to secondhand smoke

Knowledge about SHS-related health risks was greater in females of whom 63.5% had an adequate knowledge level (score ≥5) versus 49.7% among males, and the difference was statistically significant (p=0.005). Participants living with their friends had the lowest percentage of adequate knowledge (35.7%), compared to their counterparts (p=0.044). No significant association of knowledge score was observed with the other sociodemographic and lifestyle factors, notably with smoking status (p=0.181) or amount of smoking (p=0.912) (Table 4). Knowledge level was not associated with the number of persons smoking among acquaintances (p=0.392), having a smoking parent during childhood (p=0.831) and exposure by chance to SHS (p=0.220). Similarly, no association of knowledge level was observed with level of discomfort feeling (p=0.342), coping strategy (p=0.706), and attitude toward smoking prohibition in public places ( p=0.833) (Table 5).

**Table 4:** Sociodemographic and clinical and lifestyle factors associated with knowledge about secondhand smoke risks as revealed in the study of medical students of the Faculty of Medicine, King Abdulaziz University (N=416)

*Factors*	*Category*	*SHS knowledge level ^a^*				
		*Inadequate*		*Adequate*		
		*n*	*%*	*n*	*%*	*p*
**Sociodemographic**						
**Age** (years), mean ± SD **Gender**		21.77 ± 1.58		21.73 ± 1.61		0.820
	Male	92	50.3	91	49.7	
	Female	85	36.5	148	63.5	0.005*
**Marital status**	Single	173	42.5	234	57.5	
	Married	4	44.4	5	55.6	1.000
**Nationality**	Saudi	169	42.4	229	57.5	
	Non-Saudi	8	44.4	10	55.6	0.868
**Parents’ marital status**	Married	142	41.6	199	58.4	
	Divorced	17	51.5	16	48.5	
	Widow	18	42.9	24	57.1	0.548
**Rank in siblings**	Eldest	37	42.0	51	58.0	
	Not the eldest	140	42.7	188	57.3	0.914
**Monthly family income** (SAR)	<3000	5	38.5	8	61.5	
	3000–10000	30	39.0	47	61.0	
	>10000	142	43.6	184	56.4	0.730
**Residence**	Jeddah	158	41.3	225	58.7	
	Other	19	57.6	14	42.4	0.069
**Housing**	Rental	132	41.8	184	58.2	
	Own house	36	43.4	47	56.6	
	Students dormitory	9	52.9	8	47.1	0.653
**Living modality**	Alone	157	41.2	224	58.8	
	With family	2	28.6	5	71.4	
	With friends	18	64.3	10	35.7	0.044*
**Academic year**	2nd	39	41.5	55	58.5	
	3rd	15	34.9	28	65.1	
	4th	44	46.8	50	53.2	
	5th	28	38.4	45	61.6	
	6th	51	45.5	61	54.5	0.608
**GPA**	<4.5	63	38.9	99	61.1	
	3.5–4.49	107	45.9	126	54.1	
	2.5–3.49	7	33.3	14	66.7	0.259
**Lifestyle and clinical**						
**BMI** (kg/m^2^)	Underweight	20	40.8	29	59.2	
	Normal	86	40.4	127	59.6	
	Overweight	44	45.8	52	54.2	
	Obese	27	46.6	31	53.4	0.735
**Physical activity** (times per week)	0	51	41.1	73	58.9	
	1	42	48.3	45	51.7	
	2–3	16	40.0	24	60.0	
	>3	68	41.2	97	58.8	0.683
**Chronic illness**	No	16	31.4	35	68.6	
	Yes	161	44.1	204	55.9	0.085
**Smoking status**	Non-smoker	137	40.4	202	59.6	
	Quitter (>6 months)	10	52.6	9	47.4	
	Smoker	30	51.7	28	48.3	0.181
**Daily cigarettes** (among smokers)	0–3	7	53.8	6	46.2	
	4–10	5	62.5	3	37.5	
	11–20	9	50.0	9	50.0	
	>20	4	57.1	3	42.9	
	Not specified	5	41.7	7	58.3	0.912

SAR: 100 Saudi Riyal about 27 US$. GPA: grade point average. SD: standard deviation.

* Statistically significant result at p<0.05

a Inadequate score <5, adequate score ≥5.

**Table 5:** Association of knowledge with exposure and attitude to secondhand smoke among medical students of the Faculty of Medicine, King Abdulaziz University (N=416)

*Type of exposure*	*Category*	*Knowledge*		
		*Inadequate n (%)*	*Adequate n (%)*	*p*
**Number of persons smoking among acquaintances**	0	48 (46.2)	56 (53.8)	
	1	64 (38.8)	101 (61.2)	
	2	35 (44.9)	43 (55.1)	
	3	23 (50.0)	233 (50.0)	
	≥4	7 (30.4)	16 (69.6)	0.392
**Having a smoking parent during childhood**	No	70 (41.9)	97 (58.1)	
	Yes	107 (43.0)	142 (57.0)	0.831
**Exposure by chance to SHS**	No	157 (43.7)	202 (56.3)	
	Yes	20 (35.1)	37 (64.9)	0.220
**Number of places with SHS exposure**	0–1	87 (49.4)	89 (50.6)	
	2	45 (38.1)	73 (61.9)	
	3	25 (31.6)	54 (68.4)	
	≥4	20 (46.5)	23 (53.5)	0.037*
**Feeling during exposure** (discomfort score)	Good (0)	7 (50.0)	7 (50.0)	
	Not uncomfortable (1)	35 (52.2)	32 (47.8)	
	A bit uncomfortable (2)	50 (43.9)	64 (56.1)	
	Very uncomfortable (3)	78 (38.6)	124 (61.4)	
	Painful (4)	7 (36.8)	12 (63.2)	0.342
**Coping strategy**	Asked them to refrain from smoking	13 (38.2)	21 (61.8)	
	Moved away to avoid SHS	117 (44.2)	148 (55.8)	
	Did nothing	34 (38.2)	55 (61.8)	
	Smoked together	13 (46.4)	15 (53.6)	0.706
**What do you think about prohibiting smoking in public places?**	Ignoring the right to smoke is unfair	15 (38.5)	24 (61.5)	
	Better leave it to each individual’s conscience	48 (44.0)	61 (56.0)	
	It should be regulated more strictly	114 (42.5)	154 (57.5)	0.833

* Statistically significant result at p<0.05.

The significantly associated variables were entered in a logistic regression model to further investigate their independent relationships with adequate knowledge. The regression model showed that adequate knowledge about SHS was independently associated with female gender (OR=1.63; 95% CI: 1.08–2.45; p=0.019), exposure to SHS in three places (OR=2.16; 95% CI: 1.22–3.80; p=0.008), and living with friends (OR=0.43; 95 % CI: 0.19–0.99; p=0.046) (Table 6).

**Table 6:** Results of the multivariate binary logistic regression analysis of the predictors of adequate knowledge regarding SHS

*Parameter*	*Category*	*OR*	*95% CI*	*p*
**Gender**	Male	Ref.		
	Female	1.628	1.082–2.450	0.019*
**Living modality**	Alone	Ref.		
	With family	1.526	0.287–8.118	0.620
	With friends	0.432	0.189–0.987	0.046*
**Number of places with SHS exposure**	0–1	Ref.		
	2 3	1.5762.156	0.969–2.5621.224–3.799	0.0670.008*
	≥4	1.237	0.625–2.450	0.542

CI: confidence interval. OR: odds ratio.

* Statistically significant result at p<0.05.

### Association between parameters of exposure to SHS and discomfort feeling

**Figure 1:**
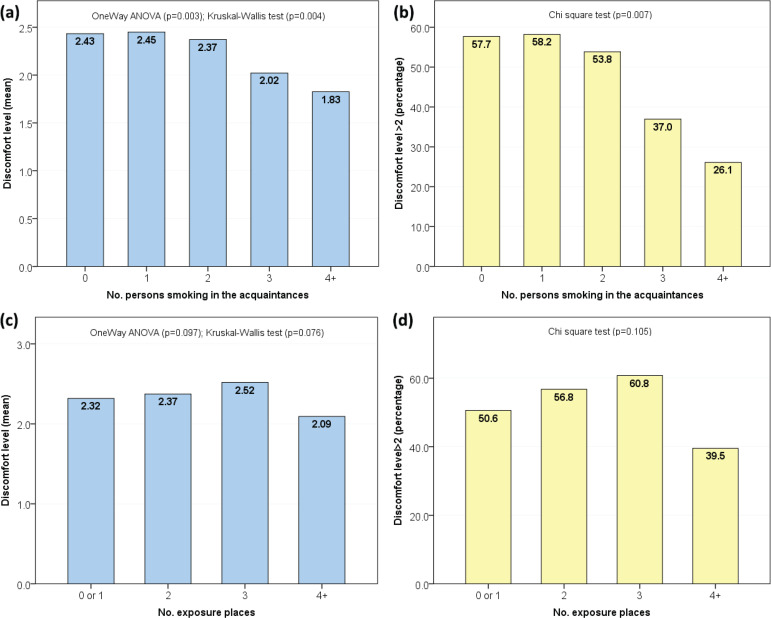
Association between parameters of exposure to SHS and discomfort feeling (a) Mean level of discomfort feeling (range: 0–4) by number of persons smoking among acquaintances. (b) Percentage of participants with significant level of discomfort (>2) by the number of persons smoking among acquaintances. (c) Mean level of discomfort feeling by number of exposure places. (d) Percentage of participants with significant level of discomfort by number of exposure places.

Both mean discomfort level (Kruskal-Wallis test, p=0.004) and percentage of participants with high level of discomfort (chi-squared, p=0.007) were inversely correlated with the number of persons smoking among acquaintances. However, no association of the discomfort level was found with the number of exposure places (Figure 1).

## DISCUSSION

In the present study, we showed that more than half of the medical students in a Saudi medical college had adequate knowledge regarding SHS risks, and that approximately 86% were exposed to SHS with no significant correlation between the level of knowledge about SHS health consequences and exposure to SHS. On the other hand, knowledge scores were significantly higher among females and lower among those living with friends compared to their counterparts. Besides, acquaintances represented the major sources of knowledge. However, levels of knowledge about SHS were not impacted by smoking status.

Knowledge regarding the risks of SHS exposure is a key factor contributing to reducing exposure to the harms of smoke. Higher knowledge levels have been associated with more protective attitudes among the general public, such as opening windows, keeping children away from the smoking environment, or adopting smoke-free homes^26,27^. In the healthcare field, knowledge levels and attitudes regarding SHS are important determinants of educating and guiding patients on the potential health risks that include all age categories; these knowledge aspects could be promoted during the early academic life of health professionals.

The results presented in our study are in agreement with other investigations. Public places in Saudi Arabia represented the most frequent sites of SHS exposure as declared by 79.8% of medical students in the present study. Similarly, 57.7% of students (n=805) at King Saud University, Riyadh, reported SHS exposure in public places^28^. Approximately 46.3% of medical students in Tabuk were exposed to SHS, and 68% emphasized the importance of implementing strategies to ban smoking in public places^29^. Furthermore, the exposure to SHS in public areas has been repeatedly demonstrated among school students and the general public^30,31^. Actually, this contradicts the national efforts that have been established in the kingdom. The World Health Organization’s global Framework Convention for Tobacco Control (FCTC) was adopted by the Ministry of Health in Saudi Arabia in 2005^32^. Consequently, smoking is banned in educational, governmental, and transportation facilities, as well as healthcare institutions and other public places. The increased exposure to SHS in these places indicates significant deficiencies in the application of tobacco laws. Therefore, there is a need to enforce these legislations and apply strict measures to reduce the burden of smoking. In addition, it is imperative to limit the access to tobacco products through the prohibition of tobacco sales, which are inherent requirements of the FCTC.

Another important element in tobacco control programs is promoting knowledge levels among the general population. Medical students are the future leaders and actors of such interventions by providing correct information; hence, their knowledge and commitment are essential pillars for the national tobacco control strategy. The mean (SD) knowledge score of medical students in the present study was 4.77 (2.01), which corresponds to a scaled score of 59.6/100. In a cross-sectional study among 420 dental students attending King Abdulaziz University, Mansour^33^ revealed a higher knowledge score (88.3/100) regarding SHS health risks. Compared to the percentage of medical students with adequate knowledge in our study (57.5%), a larger proportion (71.7%) of medical students had adequate knowledge regarding the risks of SHS in three different medical schools in the Central, Western, and Southern regions of the kingdom^18^. Beyond the discernable variances between these results and the differences in tools that were used, knowledge levels remain relatively low with regard to the studied medical student population. As a result, further interventions are required to enhance students’ knowledge and to identify the predictors of poor knowledge.

In general, it is necessary to target medical students to increase their knowledge about the risks of smoking and consequences of SHS exposure. In a case control study, a group of medical students received an educational intervention comprising online video lectures to promote their knowledge and competency to manage patients with SHSrelated disorders^34^. Although knowledge scores were generally low for all recruited participants, students who participated in the educational intervention had significant changes in their pre-test and posttest scores. Besides, the group exposed to the SHS intervention reported a significant intent to screen patients at risk in each primary care examination. In India, the implementation of a tobacco intervention training program among first year medical students promoted knowledge levels and attitudes regarding smoking cessation and SHS^35^. Indeed, this would be reflected in their perceptions about future medical practices. About 77.4% of medical students in Riyadh believed that smoking healthcare professionals are less likely to advise their patients to quit smoking^28^.

Therefore, students at medical colleges have strong perceptions about the implications of healthcare professionals as role models to guide the community and the general population. Such behavior would be further supported as the students progress to their professional life.

In our study, females had higher knowledge levels regarding SHS. Lee et al.^25^ found similar results among hospital staff in a medical institution in South Korea. Interestingly, all surveyed females in such a study were non-smokers. Females were also significantly knowledgeable in other studies conducted in Pakistan^36^ and Jordan^37^. Supposedly, females would be more aware of SHS risks because they are more concerned with the adverse effects of environmental tobacco smoke on pregnancies and fetuses^38^. This suggests that future interventions should primarily focused on male healthcare professionals and/or medical students. Notably, the influence of friends may be highlighted, which can be identified from a lower knowledge level about SHS among students living with friends (away from the family circle) as indicated in the results of the multivariate regression analysis in our study. This is further marked in the case when close friends are smokers, which is considered a major risk factor of SHS exposure outside the household^31,39^. Seemingly, male students with smoking friends have little information about SHS risks, and so they are more likely to express neutral attitudes towards smoking^40,41^. The influence of friends increases also the likelihood of future smoking, and having a non-smoking friend reduces that risk^42-44^. Cognitive vulnerabilities towards smoking uptake and SHS-related risks are influenced by peers early in children aged 9–10 years^41^. The friend effect could therefore be targeted in future intervention programs, and medical students could play a significant role to advise their friends to quit smoking.

In our study, the increased number of smokers among acquaintances has significantly reduced the participants’ discomfort levels, whereas the number of exposure places did not alter the discomfort variables. These unexpected findings may indicate an increased level of social tolerance to SHS exposure, probably leading to inability of participants to identify SHS exposure in certain instances and inaccurate reporting of SHS exposure. Another aspect of social tolerance is the findings about coping strategy showing the majority (>85%) reacting to SHS by passive avoidance or non-action, while only 8.2% declared opposing smoking in restricted places. This tolerance could be associated with high prevalence of smoking in some areas as revealed previously^45^. The impact of social tolerance is detrimental, as it causes significant harms to the exposed non-smokers and could represent a barrier to smoke-free policies. Thus, medical students should be more aware about this issue to contribute more effectively in patient education about the risks of SHS exposure, both among smokers and potential passive smokers.

### Limitations

The present study has some limitations. The crosssectional design might have resulted in a significant bias in reporting distinct variables, such as the exposure to SHS and discomfort levels, which were measured using indirect and subjective indicators. Additionally, the outcomes of our analysis might not be fully representative of other Saudi medical colleges across the kingdom. This might be compounded by the differences in medical curricula at other national colleges. However, we believe that the results of the present study could pave the way for the implementation of robust educational interventions that target future doctors. This would ultimately be reflected in the national efforts of tobacco control within healthcare institutions to promote public health awareness.

## CONCLUSIONS

Medical students in Jeddah, Saudi Arabia, had relatively low knowledge regarding the health risks of SHS exposure, particularly when compared to other medical colleges in the kingdom. While female students had higher knowledge levels than males, the friends’ circle appears to play a significant role in awareness and knowledge about the health risks of SHS. There is great interest for medical colleges to implement effective educational interventions by improving their curricula regarding the risks of SHS and the benefits of smoking cessation. Notwithstanding the efforts of the Saudi Ministry of Health to ban smoking according to the FCTC treaty, the exposure to SHS in public places is still substantial. Therefore, it is crucial to reinforce tobacco control strategies and to increase taxation of tobacco products to support local legislations.
